# Heat-Shock Protein 90 Promotes Nuclear Transport of Herpes Simplex Virus 1 Capsid Protein by Interacting with Acetylated Tubulin

**DOI:** 10.1371/journal.pone.0099425

**Published:** 2014-06-05

**Authors:** Meigong Zhong, Kai Zheng, Maoyun Chen, Yangfei Xiang, Fujun Jin, Kaiqi Ma, Xianxiu Qiu, Qiaoli Wang, Tao Peng, Kaio Kitazato, Yifei Wang

**Affiliations:** 1 Guangzhou Jinan Biomedicine Research and Development Center, National Engineering Research Center of Genetic Medicine, Jinan University, Guangzhou, PR China; 2 College of Pharmacy, Jinan University, Guangzhou, PR China; 3 State Key Laboratory of Respiratory Disease, Guangzhou Institutes of Biomedicine and Health, Chinese Academy of Sciences, Guangzhou, PR China; 4 Division of Molecular Pharmacology of Infectious Agents, Department of Molecular Microbiology and Immunology, Graduate School of Biomedical Sciences, Nagasaki University, Nagasaki, Japan; Fudan University, China

## Abstract

Although it is known that inhibitors of heat shock protein 90 (Hsp90) can inhibit herpes simplex virus type 1 (HSV-1) infection, the role of Hsp90 in HSV-1 entry and the antiviral mechanisms of Hsp90 inhibitors remain unclear. In this study, we found that Hsp90 inhibitors have potent antiviral activity against standard or drug-resistant HSV-1 strains and viral gene and protein synthesis are inhibited in an early phase. More detailed studies demonstrated that Hsp90 is upregulated by virus entry and it interacts with virus. Hsp90 knockdown by siRNA or treatment with Hsp90 inhibitors significantly inhibited the nuclear transport of viral capsid protein (ICP5) at the early stage of HSV-1 infection. In contrast, overexpression of Hsp90 restored the nuclear transport that was prevented by the Hsp90 inhibitors, suggesting that Hsp90 is required for nuclear transport of viral capsid protein. Furthermore, HSV-1 infection enhanced acetylation of α-tubulin and Hsp90 interacted with the acetylated α-tubulin, which is suppressed by Hsp90 inhibition. These results demonstrate that Hsp90, by interacting with acetylated α-tubulin, plays a crucial role in viral capsid protein nuclear transport and may provide novel insight into the role of Hsp90 in HSV-1 infection and offer a promising strategy to overcome drug-resistance.

## Introduction

Herpes simplex virus type 1 (HSV-1) is a member of the Herpesviridae family [1]. The HSV-1 virion consists of a relatively large, double-stranded, linear DNA genome encased within an icosahedral protein cage called the capsid [2]. HSV-1 has mainly oral and ocular manifestations, and after primary infection, the virus can establish latency in the trigeminal or cervical ganglia. The latent virus can then be reactivated to induce neurite damage and neuronal death. The currently available anti-HSV drugs are mainly nucleoside analogs, such as acyclovir (ACV), and all of them target viral DNA replication. However, drug-resistant HSV strains, and particularly ACV-resistant HSV strains, emerge frequently [3], [4]. Therefore, the development of new anti-HSV agents with different mechanisms of action is a matter of great urgency.

Rapid progress has been achieved based on a deep understanding of the molecular mechanisms involved in different phases of the HSV-1 life cycle [3]. After entering into the cytoplasm, nuclear targeting of incoming viruses depends on the cellular cytoskeleton-mediated transport system [5]. Actin filaments play a crucial role for short-range movement and viral penetration or endocytosis [6], whereas microtubules (MTs) provide tracks for the long-distance transport of endocytic/exocytic vesicle because of the directionality of MTs [7]. Incoming HSV-1 particles are transported along MTs to the nucleus via interactions with an MT-dependent cellular molecular motor known as the cytoplasmic dynein/dynactin complex. Given that most of the tegument is lost during entry or stays in the cytoplasm, the viral protein(s) that are candidates for directly engaging dynein/dynactin include the remaining inner tegument and capsid proteins. Although MTs enable the proper movement of cytosolic capsids into the nucleus [7], further details regarding viral intracellular translocation remain unknown.

Heat shock protein 90 (Hsp90) is a highly conserved molecular chaperone that plays essential roles in constitutive cell signaling and adaptive responses to stress, such as microbial infection [8]. Hsp90 accounts for 1–2% of the total protein in unstressed cells, and in mammals, there are two cytoplasmic Hsp90 isoforms, the stress induced Hsp90α and the constitutively expressed Hsp90β, as well as an ER resident homologue Grp94 (also called gp96), and a mitochondrial variant, TRAP1 [9]. Additionally, Hsp90 has been shown to be important for many different viruses that require chaperone functions for viral protein folding, replication, transport, and assembly [10]. In fact, the dependence of viruses on Hsp90 appears to be nearly universal. Strikingly, for viruses tested to date, replication appears to be sensitive to Hsp90 inhibitors at concentrations not affecting cellular viability [11]. Geldanamycin (GA), an Hsp90 inhibitor, can inhibit the replication of HSV-1 [12]. In our previous studies [13], [14], we reported the *in vitro* and *in vivo* anti-HSV activity of 2-aminobenzamide derivatives, including BJ-B11, SNX-25a, SNX-2112, and SNX-7081, which are all Hsp90 inhibitors. These inhibitors displayed significant efficacy against herpes simplex keratitis in a rabbit model and mainly exerted antiviral effects in the early stage of infection. However, the underlying mechanism of action has not been determined to date.

In the present study, we found that HSV-1 infection stimulates upregulation and nuclear translocation of Hsp90, which coincide with the enhanced acetylation of α-tubulin and the nuclear transport of the viral capsid protein ICP5. We also revealed that inhibition of Hsp90 prevents ICP5 nuclear transport and tubulin acetylation. Furthermore, Hsp90 inhibitors demonstrated potent antiviral effects against a drug-resistant HSV-1 strain and a laboratory strain. This study provides novel insight into the mechanisms of Hsp90 action that are involved in HSV-1 early infection and offering a promising strategy against drug-resistant HSV-1 infection.

## Materials and Methods

### Cells and Viruses

MRC-5 cells (ATCC) and Vero cells (ATCC) were cultured as described previously [15]. All experiments were performed with the HSV-1 strain F (ATCC), a kind gift from Hong Kong University. The clinical-isolated ACV-resistant HSV-1 strain (named C106) used in this work was obtained from the Guangzhou Institutes of Biomedicine and Health [16].

### Compounds, Antibodies, Reagents, and Plasmids

BJ-B11 was synthesized according to previously reported methods [17]. ACV and 17-AAG were purchased from Alexis Biochemicals. The primary antibodies used in this work are as follows: mouse monoclonal antibody (mAb) against the HSV-1+ HSV-2 ICP5 major capsid protein (Abcam), a mouse mAb against the HSV-1 ICP8 major DNA-binding protein (Abcam), a mouse mAb against the HSV-1+ HSV-2 ICP27 protein (Abcam), an anti-Hsp90 rabbit mAb (Cell Signaling Technology), an anti-Hsp90 mouse mAb (Santa Cruz), an anti-α-tubulin rabbit mAb (Cell Signaling Technology), an anti-acetyl-α-tubulin Lys40 rabbit mAb (Cell Signaling Technology), and an anti-GAPDH rabbit mAb (Cell Signaling Technology). The secondary antibodies included Alexa Fluor 488-conjugated goat anti-mouse or anti-rabbit IgG (H+L) (Invitrogen), Alexa Fluor 647-conjugated goat anti-mouse IgG (H+L) (Invitrogen), horseradish peroxidase (HRP)-conjugated anti-mouse/rabbit IgG (GE Healthcare). For immunoprecipitation, Protein A/G PLUS-Agarose (Santa Cruz) and normal mouse/rabbit IgG (Santa Cruz) were used.

To construct an Hsp90α overexpression vector (pEGFP-Hsp90α), the human Hsp90α coding sequence without the TGA stop codon was cloned from the total cDNA of MRC-5 cells with the forward primer 5′-AAA ACT GCA GAT GCC TGA GGA AAC CCA GAC-3′ and the reverse primer 5′-CGG GGT ACC TCTA CTT CTT CCA TGC GTG-3′. The PCR fragment was then digested with Pst I (Takara) and Kpn I (Takara) and inserted into the multiple cloning site of the pEGFP-N1 plasmid (Clontech) under the control of the cytomegalovirus (CMV) promoter. The recombinant pEGFP-Hsp90α vector was successfully constructed, as confirmed by DNA sequencing analysis.

### Plaque Assay

Plaque assay was used to determine the virus titers [15]. Briefly, Vero cells were seeded into 12-well plates before the cells were incubated with virus suspension. After appropriate time the virus inoculum was then removed and the overlay medium (DMEM containing 2% FBS and 1% methylcellulose) was added. After an additional 72 h of incubation, the cell monolayers were fixed with 10% formalin and stained with 1% crystal violet. The plaques were counted, and the virus titers were calculated.

### Antiviral Activity Assay

MTT assay was first used to assess the cytotoxicity of compounds [18]. The 50% cytotoxic concentration (CC_50_) was calculated from three independent experiments and expressed as the mean ± SD. Then anti-HSV-1 activity of the compounds was evaluated by a virus-induced CPE inhibitory assay. A plaque reduction assay was also used to evaluate the HSV-1 inhibition activity of compounds. According to the observed number of plaques, the IC_50_ value was defined as the minimal concentrations of each tested compound that was required to reduce the plaque number by 50% and was calculated as previously described [19].

### Transfection

The transfection of MRC-5 cells with the pEGFP-Hsp90α or pEGFP-N1 plasmid was performed with Lipofectamine LTX and PLUS reagents (Invitrogen). For a 24-well transfection, 1.5 µg of the pEGFP-Hsp90α or pEGFP-N1 plasmid was used. SiRNA transfection was conducted using Lipofectamine RNAiMax reagent (Invitrogen). The Hsp90α-siRNA duplex consisted of oligonucleotides with the sequences 5′-CUA CAA UUC CUC UGA UAA U-3′ and 5′-AUU AUC AGA GGA AUU GUA G-3′. A scrambled siRNA duplex of the oligonucleotides 5′-UUC UCC GAA CGU GUC ACG UTT-3′ and 5′-ACG UGA CAC GUU CGG AGA ATT-3′, which do not target any gene product, was used as a negative control. All siRNAs were obtained from Sigma-Aldrich. For a 24-well transfection, 1 µg of RNAi duplex was used and at 48 h post-transfection, the cells were infected with HSV-1 for further studies.

### Real-time Fluorescent Quantitative RT-PCR

The total RNAs of cells infected with HSV-1 (MOI = 10) for different times in the presence of 0.8 µM Hsp90 inhibitor were extracted (TRIzol reagent from Invitrogen) and reverse transcribed (PrimeScript RT reagent Kit, Takara) according to the manufacture. Then the mRNA expression levels were determined and analyzed using Bio-Rad CFX96 real-time PCR system (Bio-Rad) [14]. The mRNA expression levels were normalized to GAPDH expression. The primer pairs used were as follows: HSV-1 UL54 F (5′-TGG CGG ACA TTA AGG ACA TTG-3′), UL54 R (5′-TGG CCG TCA ACT CGC AGA-3′), HSV-1 UL29 F (5′-AGC TCG TCC GTG TAC GTC TT-3′), UL29 R (5′-CCC TCG GTA ACG ACC AGA TA-3′), HSV-1 UL27 F (5′-GCC TTC TTC GCC TTT CGC-3′), UL27 R (5′-CGC TCG TGC CCT TCT TCT T-3′), Hsp90α F (5′-ACA GGG TCT CAC TCT GTC G-3′), Hsp90α R (5′-GGA AGG ATA GCA GTG TTA GG-3′), GAPDH F (5′-CCC ACT CCT CCA CCT TTG AC-3′), and GAPDH R (5′-TCT TCC TCT TGT GCT CTT GC-3′).

To detect gene expression after Hsp90α siRNA or pEGFP-Hsp90α plasmid transfection, MRC-5 cells grown in 24-well culture plates were first transfected with Hsp90α siRNA or scrambled siRNA or the pEGFP-Hsp90α or pEGFP-N1 plasmids. The cells were then infected with HSV-1 (MOI = 10), treated with Hsp90 inhibitors at 48 h post-transfection, harvested, and analyzed for gene expression, as described above.

### Laser Scanning Confocal Immunofluorescence Microscopy

To quantify HSV-1 capsid protein ICP5 trafficking to the nuclear, MRC-5 cells grown in 25-cm^2^ culture flasks were transfected with the pEGFP-Hsp90α or pEGFP-N1 plasmid or treated with 0.8 µM BJ-B11 and infected with HSV-1 (MOI = 10) for different lengths of time. The samples were processed as described before [17]. Briefly, after fixing, permeabilizing and blocking, the cells were immunostained with a primary antibody against HSV-1 ICP5 (1∶3000) for 1 h, and the cells were then probed with an Alexa Fluor 488-conjugated anti-mouse antibody (1∶1000) for another 1 h. Additionally, 1 mg/ml DAPI (Biotium) and 5 µM TRITC-phalloidin (Sigma-Aldrich) were added to label nuclei (15 min) and F-actin (40 min), respectively. Finally, fluorescence images were captured with a confocal laser scanning microscope (Zeiss). In addition, each dish was acquired at least five fields of view for the purpose of counting the number of ICP5 associated nuclei (here defined as positive nuclei), percentage of which was calculated to evaluate the effect of Hsp90 inhibitors on virus entry and intracellular migration.

For total Hsp90 and HSV-1 ICP5 observation, MRC-5 cells were infected with HSV-1 (MOI = 10) for 4 h in the presence of 0.8 µM Hsp90 inhibitor. The cells were then stained with anti-ICP5 antibody and an Alexa Fluor 647-conjugated anti-mouse antibody (1∶1000). For anti-Hsp90 antibody (1∶500) staining, an Alexa Fluor 488-conjugated secondary antibody (1∶1000) was used.

To observe the relationship between Hsp90 and acetyl-α-tubulin, MRC-5 cells were stained with anti-acetyl-α-tubulin (1∶1000) antibody and Alexa Fluor 488-conjugated secondary antibody (1∶1000). An Alexa Fluor 647-conjugated secondary antibody (1∶1000) was used for anti-Hsp90 antibody (1∶500) staining.

### Western Blotting

MRC-5 cells infected with HSV-1 (MOI = 10) for different lengths of time in the presence or absence of inhibitors were lysed in RIPA buffer (Beyotime, China) and separated by 6–15% gradient SDS-PAGE. Then the samples were transferred to nitrocellulose and incubated with primary and HRP-conjugated secondary antibodies. Interested proteins were detected by enhanced chemiluminescence (Beyotime, China). The band intensity of each protein was calculated using Image J software and normalized to GAPDH. The fold change of each protein was compared with the cell control.

### Co-immunoprecipitation (Co-IP)

MRC-5 cells were treated with Hsp90 inhibitor (0.8 µM) and infected with HSV-1 (MOI = 10) for 4 h. The cells were then lysed and the protein concentrations were measured and adjusted to 1 mg/mL. The lysate was precleared by adding 1.0 µg of the appropriate control IgG (normal mouse or rabbit IgG, corresponding to the host species of the primary antibody), together with 20 µL of resuspended volume of Protein A/G PLUS-Agarose. Afterwards, the mixture was incubated at 4°C for 30 min. The optimal dilution of primary antibody was added to the cell lysates (supernatant), incubated for 1 h at 4°C, and then incubated at 4°C overnight with 20 µL of resuspended volume of Protein A/G PLUS-Agarose. Next, the immunoprecipitates were collected, washed with PBS, and resuspended in 40 µL 1× SDS-PAGE buffer (Beyotime, China). The samples were boiled for 2–3 min and analyzed by Western blotting and autoradiography, and total-protein samples were used as the input control.

## Results

### Hsp90 Inhibitors Exhibit Potent Inhibitory Activity against a Drug-resistant HSV-1 Strain

First the antiviral effect of BJ-B11, a novel Hsp90 inhibitor [20], the representative Hsp90 inhibitor 17-N-allylamino-17-demethoxygeldanamycin (17-AAG) and ACV, which served as positive controls representing an anti-HSV-1 drug was determined (Table 1). Both BJ-B11 and 17-AAG exhibited significant inhibitory activity more potent than that of ACV against ACV-resistant strain. BJ-B11 was less cytotoxic than 17-AAG and more potent in inhibiting HSV-1 replication.

**Table 1:** Cytotoxicity, anti-HSV activity, and therapeutic index of Hsp90 inhibitors.

Compound	Cytotoxicity a (CC_50_, µM)	Anti-HSV-1 F strain activity		Anti-ACV-resistant HSV-1 strain activity	
		IC_50_ b (µM)	TI c	IC_50_ b (µM)	TI c
BJ-B11	65.20±4.64	0.36±0.27	181.1	0.30±0.25	217.3
17-AAG	16.71±2.50	0.52±0.30	32.1	0.45±0.31	37.1
ACV	>200	0.90±0.38	>222	>200	/

Note: The values are the mean ± SD of three independent experiments.

a The cytotoxic effect was determined by the MTT assay. CC_50_ was defined as the concentration reducing cell viability by 50%.

b The antiviral activity was determined by the plaque reduction assay. IC_50_ was the concentration that inhibited 50% of HSV replication.

c The therapeutic index (TI) was defined as the ratio of CC_50_ to IC_50_.

### Hsp90 Inhibitors Exhibit Antiviral Activity Mainly in the Early Stage of Infection

**Figure 1:**
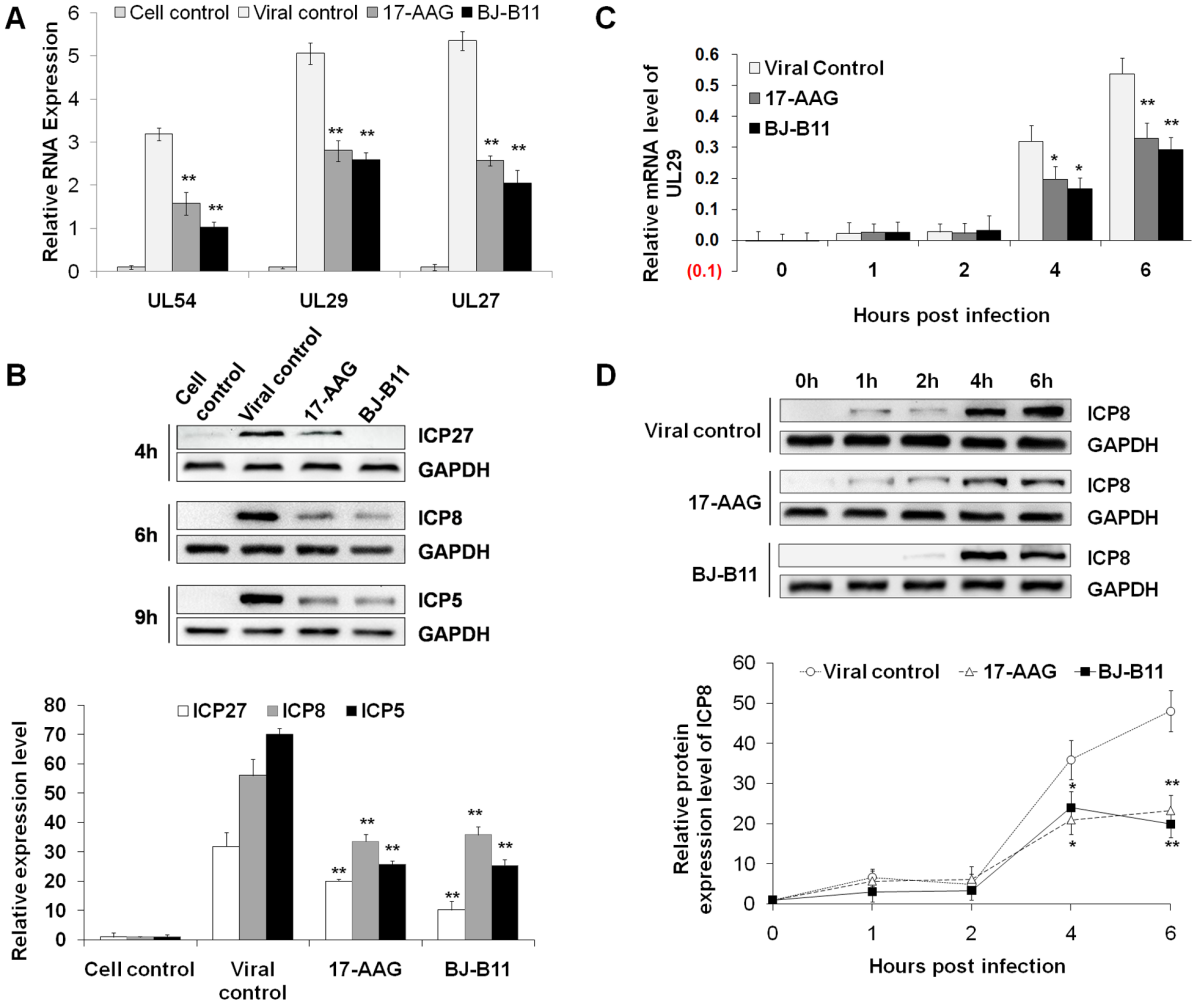
Hsp90 inhibitors suppress viral RNA synthesis and protein expression. (**A**) Inhibition of viral RNA synthesis. MRC-5 cells were infected with HSV-1 (MOI = 10) in the presence of Hsp90 inhibitor (0.8 µM). RNA samples were extracted at 4, 6, and 9 h p.i. and reverse transcribed to cDNA, which was used for UL54 (immediate early gene), UL29 (early gene), and UL27 (late gene) detection, respectively. (**B**) Inhibition of viral protein expression. MRC-5 cells were infected with HSV-1 (MOI = 10) in the presence of Hsp90 inhibitor (0.8 µM). Protein samples were extracted at 4, 6, and 9 h p.i. and used for ICP27 (immediate early protein), ICP8 (early protein), and ICP5 (late protein) detection, respectively. The Western blotting results shown in the bar graph were normalized to GAPDH expression and were expressed as the fold increase relative to the cell control. (C, D) Time-dependent inhibition of viral RNA synthesis or protein expression. MRC-5 cells were infected with HSV-1 for indicated times in the presence of Hsp90 inhibitor (0.8 µM). Total RNA or protein was extracted and analyzed for UL29 (C) and ICP8 expression (D). The results were expressed as the fold increase relative to the cell control. Each value represents the mean ± SD of three independent experiments (*, *P*<0.05; and **, *P*<0.01, compared with the viral control).

We also confirmed the antiviral effects of Hsp90 inhibitors on HSV-1 replication. MRC-5 cells were infected with HSV-1 in the present of Hsp90 inhibitors (0.8 µM), and total RNA samples were extracted at 4, 6 and 9h post-infection (p.i.), and viral UL54 (Immediate early gene), UL29 (Early gene) and UL27 (Late gene) were assayed by quantitative real-time PCR, respectively (Fig. 1A). Expressions of all viral genes were significantly suppressed by treatment of Hsp90 inhibitors. Down-regulation of the immediate early gene expression suggested that viral nuclear trafficking may be inhibited. Besides, the expressions of ICP27 (Immediate early protein), ICP8 (Early protein) and ICP5 (Late protein) were also reduced in the presence of Hsp90 inhibitors (Fig. 1B). To further identify the time point of Hsp90 inhibitor action, we detected the expression of both an early gene (UL29) and an early protein (ICP8) at different time point p.i. (Fig. 1C and 1D). Compared with the viral control, the expression of UL29 and ICP8 was significantly reduced by Hsp90 inhibitors from 4 h p.i. These results indicated that BJ-B11 and 17-AAG exhibit antiviral activity mainly in the early stage of infection.

### Nuclear Transport of Hsp90 Coincides with Viral Capsid Protein ICP5 at the Early Stage of HSV-1 Infection

**Figure 2:**
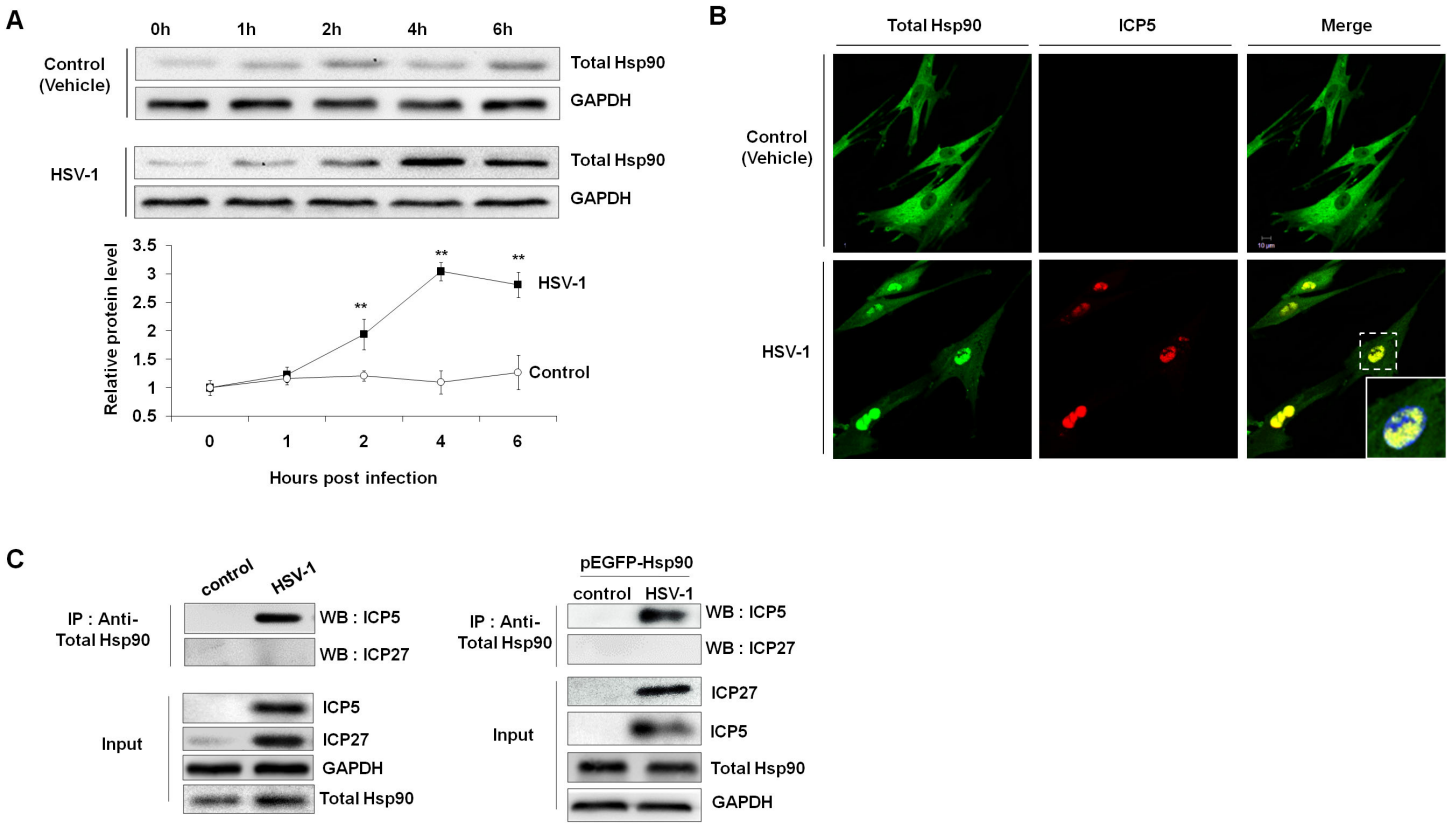
HSV-1 infection induces Hsp90 upregulation and nuclear translocation. (A) MRC-5 cells were infected with HSV-1 (MOI = 10) for 0, 1, 2, 4, or 6 h. The cells were then harvested, lysed, and analyzed for total Hsp90 expression. The Western blotting results shown in the line graph were normalized to GAPDH expression and were expressed as the fold increase relative to the cell control. Each value represents the mean ± SD of three independent experiments. (B) MRC-5 cells infected with HSV-1 (MOI = 10) for 4 h were fixed, permeabilized and stained for ICP5 (red), total Hsp90 (green), and nuclei (blue). (C) Interaction between ICP5 and Hsp90. Cells transfected with or without pEGFP-Hsp90 were lysed, immunoprecipitated with anti-Hsp90 antibody and probed with indicated antibodies. Non-capsid protein ICP27 was used as a negative control.

To define the role of Hsp90 in HSV-1 infection, we first examined the expression of total Hsp90 protein at different time points after infection (Fig. 2A). In HSV-1-infected cells, Hsp90 was significantly upregulated at 4 hours and 6 hours p.i. Subcellular localization of Hsp90 at 4 h p.i. was also analyzed using laser scanning confocal immunofluorescence microscopy (Fig. 2B). In uninfected cells, the Hsp90 protein was predominantly diffuse in the cytoplasm, whereas Hsp90 was enriched in the nucleus of HSV-1-infected cells. We further study the major viral capsid protein ICP5 with immunofluoresence staining. ICP5 is a viral late gene-encoded protein that synthesized mainly at late times (>12 h p.i.) during HSV-1 infection and at an early time (<6 h p.i.) the ICP5 dot in the cytoplasm can represent incoming virions. We found that Hsp90 and ICP5 were co-localized to the nucleus of infected cells at 4 h p.i. In addition, co-immunoprecipitation (co-IP) experiment was performed to confirm the interaction between Hsp90 and ICP5 (Fig. 2C). Apparently, there was a significant interaction between ICP5 and Hsp90 but no interaction between ICP27 (non-capsid protein) and Hsp90 was observed. Besides, by over-expressing of GFP-fused Hsp90 in cells, we still found that GFP-Hsp90 was directly associated with viral ICP5 protein. These results demonstrated that HSV-1 infection induces Hsp90 upregulation and nuclear translocation, which interacts with ICP5, suggesting Hsp90 is involved in the nuclear transport of viral capsid protein ICP5.

### Hsp90 Interacts with Acetylated α-tubulin

**Figure 3:**
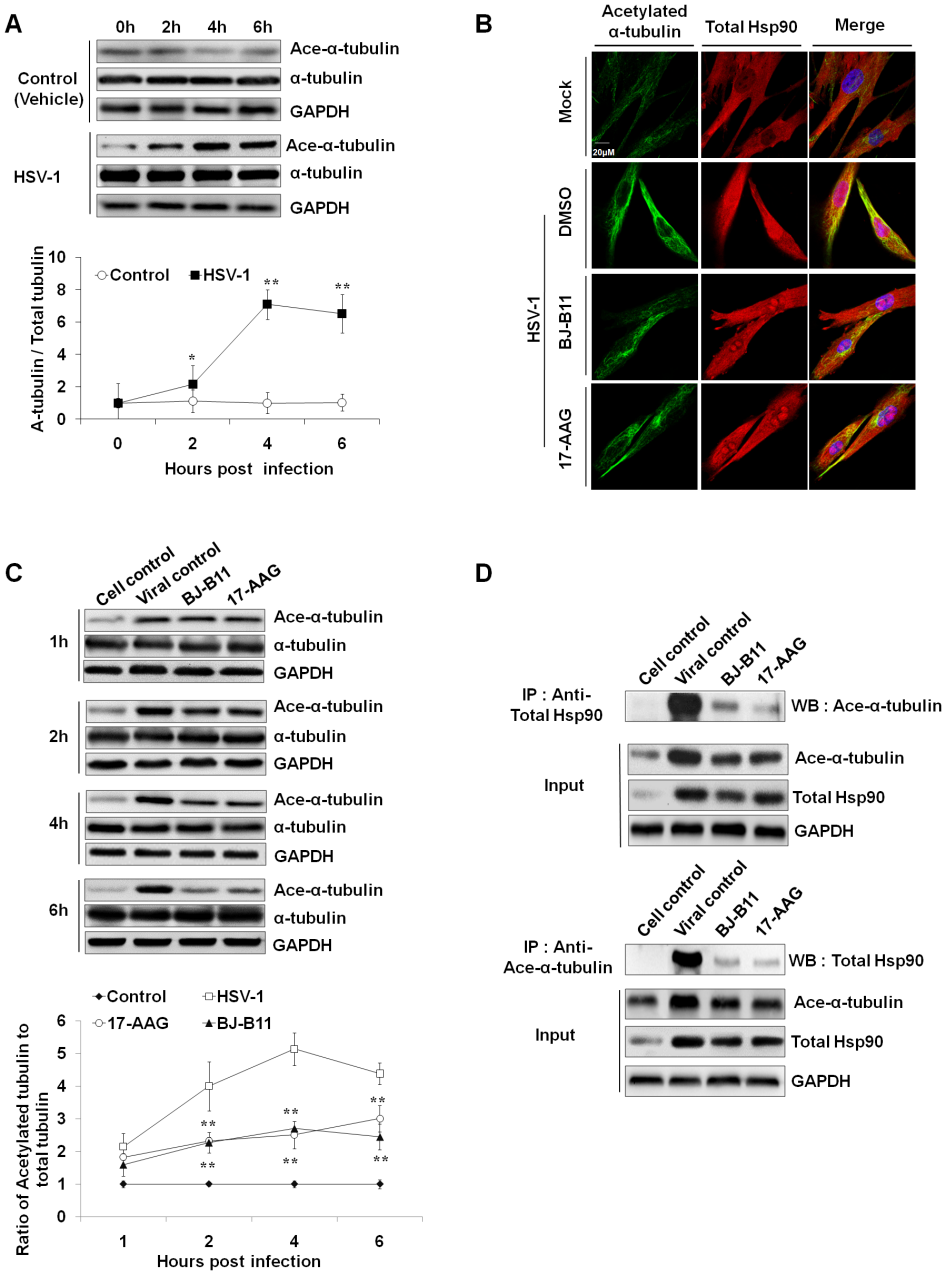
HSV-1 infection facilitates α-tubulin acetylation which is inhibited by Hsp90 inhibition. (A) Western blotting shows HSV-1 infection enhanced acetylation of α**-**tubulin. (B) Colocalization between Hsp90 and acetylated α-tubulin is reduced by Hsp90 inhibition. MRC-5 cells were infected with HSV-1 (MOI = 10) for 4 h in the presence or absence of Hsp90 inhibitors. The cells were then fixed, stained for acetylated α-tubulin (green), total Hsp90 (red), and nuclei (blue). (C, D) Hsp90 inhibitors (0.8 µM) inhibit HSV-1-induced acetylation of α-tubulin (C) and the interaction between Hsp90 and acetylated α-tubulin (D). Co-IP experiment shows a reduced interaction between Hsp90 and α-tubulin. Each value represents the mean ± SD of three independent experiments (*, *P*<0.05; and **, *P*<0.01, compared with the viral control).

Recent reports have shown that microtubule-disrupting drugs strongly reduced the transport of HSV-1 capsids to the nucleus [21] and acetylated tubulin can enhance MT-binding and transport of the motor protein kinesin-1 [22]. Influenza A virus infection enhances the acetylation of tubulin [23], thus prompting us to examine whether HSV-1 infection can induce MT acetylation. Apparently, the expression of acetylated α-tubulin in infected cells increased beginning at 2 h p.i., reached a maximum at 4 h p.i., and slightly decreased at 6 h p.i. (Fig. 3A). The kinetics of tubulin acetylation correlated well with the kinetics of Hsp90 upregulation, suggesting that Hsp90 might be involved in acetylation of α-tubulin. In addition, previous studies have demonstrated that Hsp90 binds MTs and is involved in the reorganization of the microtubular network [24]. Thus we tested the correlation between Hsp90 and tubulin (Fig. 3B) [25]. The colocalization of Hsp90 with acetylated α-tubulin and enhanced immunofluorescence were observed in HSV-1-infected cells while such colocalization was suppressed in the inhibitor-treated groups. Besides, Hsp90 inhibitors significantly decreased the level of acetylated α-tubulin beginning at 2 h p.i. (Fig. 3C). Furthermore, co-IP experiment also demonstrated a reduced interaction between acetylated α-tubulin and Hsp90 in the presence of Hsp90 inhibitors (Fig. 3D). Taken together, these results demonstrated that Hsp90 inhibitors suppressed the HSV-1-induced acetylation of α-tubulin, and the interaction of Hsp90 with MTs.

### Inhibition of Hsp90 Reduces Nuclear Transport and Expression of Viral Capsid Protein

**Figure 4:**
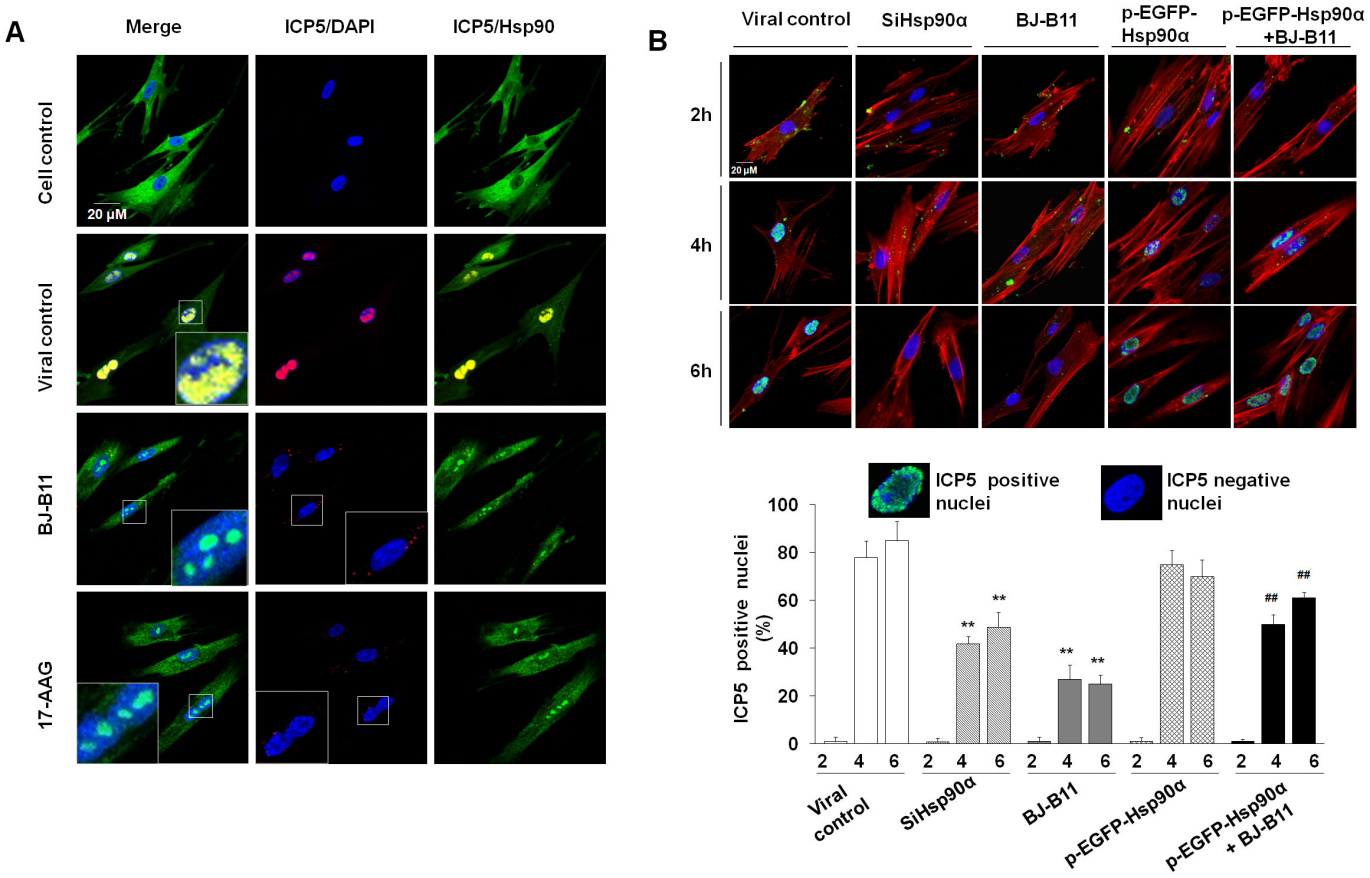
Hsp90 plays a crucial role in ICP5 nuclear translocation. (A) Confocal images show capsid protein transport reduced by Hsp90 inhibition. MRC-5 cells exposed to HSV-1 (MOI = 10) for 4 h under the treatment of Hsp90 inhibitor (0.8 µM) were stained for ICP5 (red), total Hsp90 (green), and nuclei (blue). (B) Hsp90 is important for capsid protein nuclear transport. Cell monolayers infected with HSV-1 (MOI = 10) for different times were stained for ICP5 (red), total Hsp90 (green), and nuclei (blue). Five images per dish were acquired by LSM for counting of ICP5 docked in nuclear. The percentage of positive nuclei (nuclei with ICP5) was calculated. Each value represents the mean ± SD of three independent experiments (**, *P*<0.01, compared with the viral control. ^##^, *P*<0.01, compared with the BJ-B11-treated group).

Considering that tubulin acetylation can stabilize microtubule and thereby promote viral nuclear translocation, it is easy to envision that Hsp90 inhibitors may interrupt HSV-1 capsid protein (e.g. ICP5) nuclear transport by reducing the acetylation of tubulin and its interaction with Hsp90. To confirm the role of Hsp90 in nuclear transport of viral capsid protein, first we confirmed the subcellular localization of these proteins using laser scanning microscopy (LSM) (Fig. 4A). At 4h p.i., newly ICP5 has not been synthesised and thereby those ICP5 dots in the cytoplasm represent incoming virions. Compared with the viral control, in which ICP5 and Hsp90 were highly concentrated in nucleus, accumulation of Hsp90 in nucleus was reduced and Hsp90 formed punctate domains in the inhibitor-treated cells. In contrast, ICP5 was dispersed in the cytoplasm in the presence of inhibitors, suggesting that Hsp90 inhibition significantly prevented nuclear transport of viral capsid protein ICP5. We also modulated Hsp90 expression by siRNA or overexpression to confirm the role of Hsp90 in ICP5 nuclear transport (Fig. 4B). At 4h p.i. and 6h p.i., ICP5 speckles were mostly enriched in nucleus in the viral control group, in contrast, no ICP5 speckles were observed in the nuclei of Hsp90 inhibitor-treated group or Hsp90α siRNA-treated group, in which the viral ICP5 protein were still distributed in the cytoplasm. Besides, at 6h p.i., ICP5 was hard to detect in cells and might be degraded (Fig. 4B). However, nuclear accumulation of ICP5 in the pEGFP-Hsp90α transfection group was similar to that in the viral control group, suggesting overexpression of pEGFP-Hsp90 restored the ICP5 nuclear transport that was inhibited by BJ-B11 treatment. Furthermore, the percentage of ICP5-positive nuclei was calculated to evaluate the efficiency of ICP5 nuclear transport (Fig. 4B). A statistical analysis of the data showed that Hsp90 is significantly associated with efficient viral ICP5 nuclear transport. Those results indicated that Hsp90 promotes nuclear transport of viral capsid protein and inhibition of Hsp90 may lead to degradation of capsid protein.

**Figure 5:**
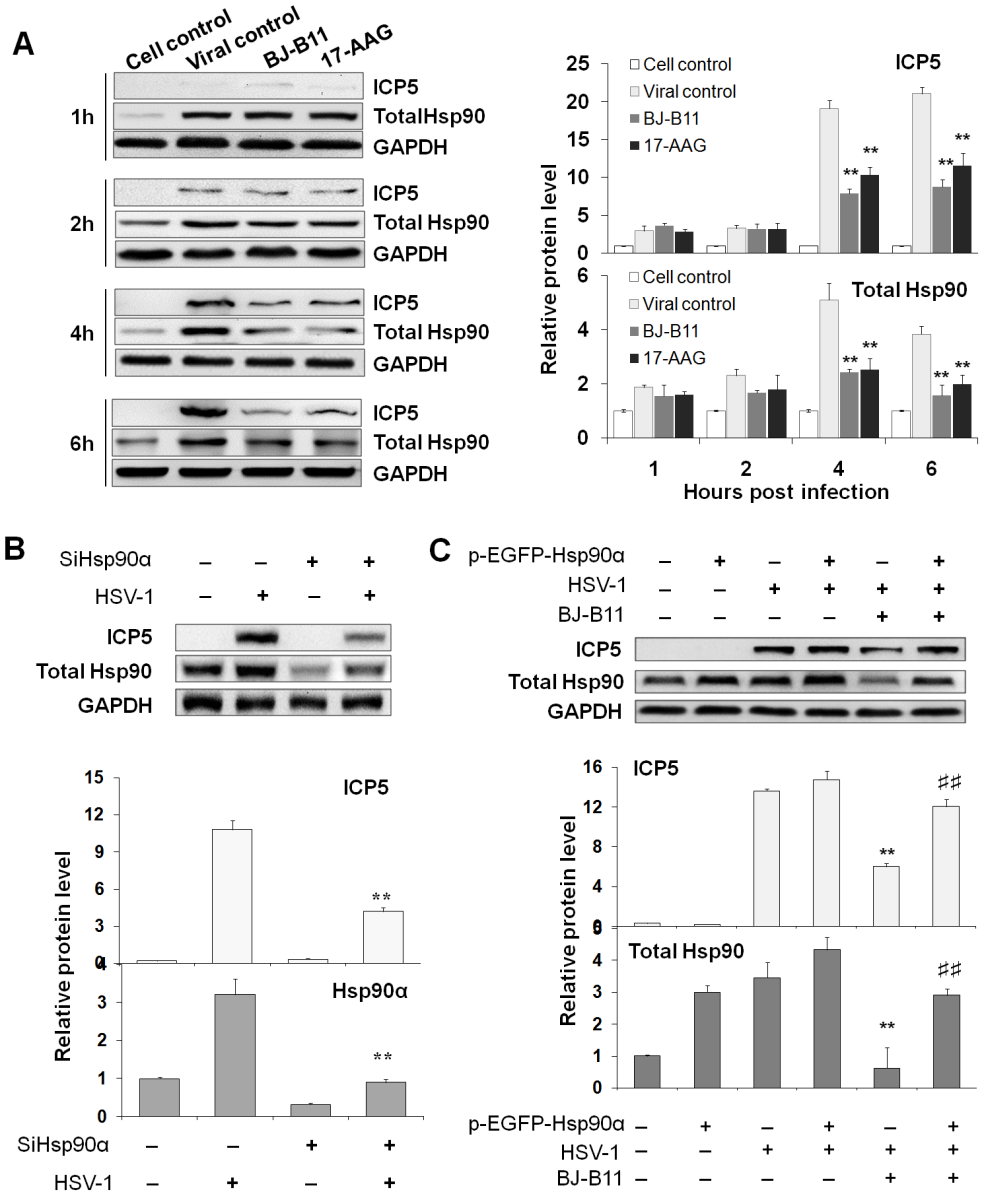
Inhibition of Hsp90 reduces ICP5 expression. (A) MRC-5 cells infected with HSV-1 (MOI = 10) for 1, 2, 4, or 6 h in the presence of Hsp90 inhibitors (0.8 µM) were harvested, lysed, and analyzed by Western blotting for ICP5 and total Hsp90 expression. The Western blotting results shown in the bar graph were normalized to GAPDH expression and were expressed as the fold increase relative to the cell control. (B) Western blot analysis of the expression of ICP5 and Hsp90 in cells transfected with Hsp90 siRNA. The Western blotting results shown in the bar graph were normalized to GAPDH expression and were expressed as the fold increase relative to the cell control. (C) Overexpression of Hsp90 restores the expression level of ICP5. MRC-5 cells transfected with the pEGFP-Hsp90α or pEGFP-N1 plasmid were infected with HSV-1 (MOI = 10) and treated with BJ-B11 (0.8 µM). (**, *P*<0.01, compared with the viral control. ^##^, *P*<0.01, compared with the BJ-B11-treated group).

Then to confirm whether the chaperone activity of Hsp90 is required for viral capsid protein ICP5 expression, Hsp90 inhibitor 17-AAG and BJ-B11 were used to treat HSV-1-infected cells, and the expression level of ICP5 and Hsp90 at different time points after infection was measured respectively (Fig. 5A). During HSV-1 infection, more and more virions entered cells and the level of ICP5 increased. Treating the infected cells with Hsp90 inhibitors significantly reduced the level of both ICP5 and Hsp90 beginning at 4 h p.i. Modulation of Hsp90 expression also affects ICP5 expression. As shown in Fig.5B **,** Hsp90 knockdown by siRNA efficiently reduced Hsp90 expression level and significantly decreased the expression of ICP5 protein in infected cells. In addition, overexpression of Hsp90 protein can counteract the effect of Hsp90 inhibitors (Fig. 5C). Overexpression of Hsp90 slightly enhanced the expression level of ICP5 at 4 h p.i. and restored ICP5 protein expression, which was decreased by BJ-B11 treatment, suggesting that Hsp90 is crucial for viral capsid protein expression.

### Hsp90 Inhibitors Inhibit ICP5 Nuclear Translocation of ACV-resistant Virus

**Figure 6:**
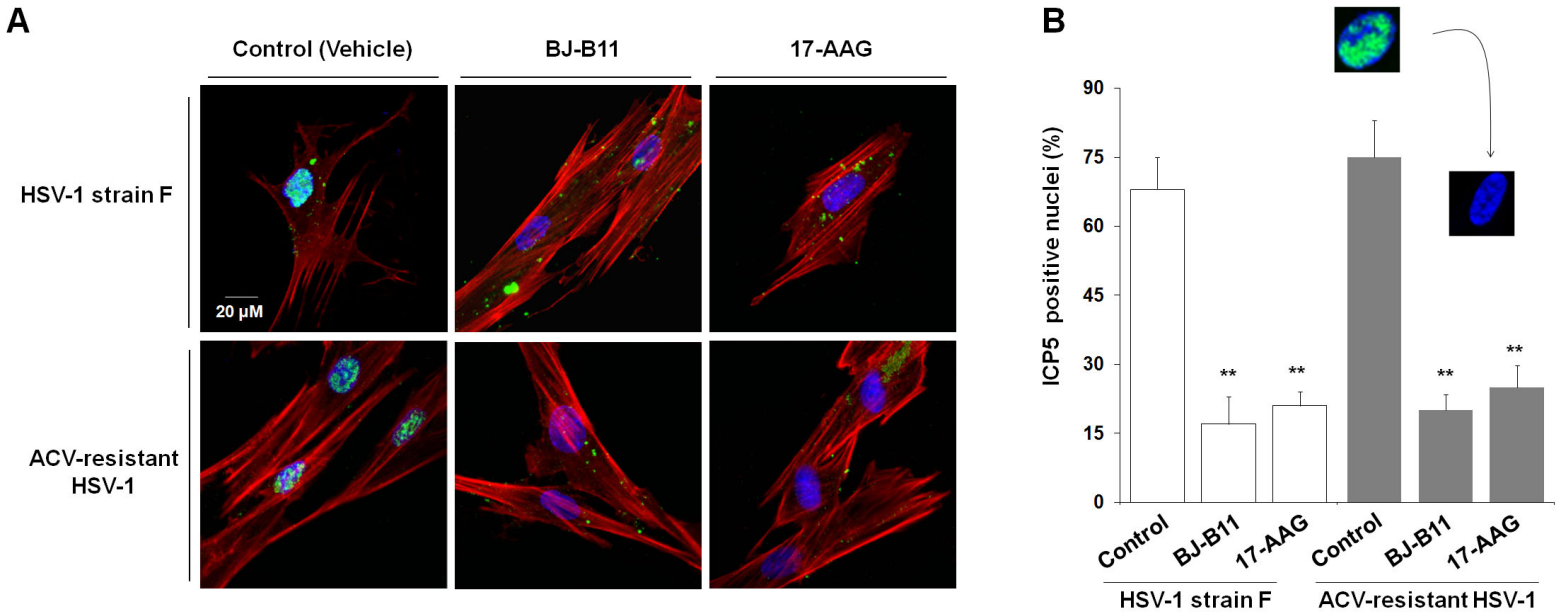
Hsp90 inhibitors suppress intracellular translocation of ACV-resistant virus capsid protein ICP5. (A) Effects of Hsp90 inhibitors on ICP5 transport. After 4h infection in the presence of BJ-B11 or 17-AAG, the cells were fixed and confocal images showed the inhibition of capsid transport ether in HSV-1 F strain or in ACV-resistant HSV-1. (B) Quantification of ICP5-positive nuclei. Each value represents the mean ± SD of three independent experiments (**P<0.01, compared with the HSV-1 F strain control, or the ACV-resistant HSV-1 strain control, respectively).

To monitor the effect of Hsp90 inhibitor treatment on the nuclear translocation of viral ICP5 protein, we observed the subcellular localization of the major viral capsid protein ICP5 (Fig. 6A) and calculated the percentage of ICP5-positive nuclei (Fig. 6B). At 4 h p.i., the viral capsids were mostly enriched in the nucleus in the viral control group, but not in the presence of Hsp90 inhibitors, in which the ICP5 protein were still distributed in the cytoplasm. A statistical analysis of the data showed that the percentage of ICP5-positive nuclei was approximately 70% and 20% for the viral control and the Hsp90 inhibitor-treated groups at 4 h p.i., respectively. These results indicated that Hsp90 inhibitors suppress the ICP5 nuclear transport of the ACV-resistant HSV-1 strain as well as the F strain during the early stage of infection.

## Discussion

Previous studies have demonstrated that capsid nuclear transport of HSV-1 was dependent on an intact microtubule network and cytoplamic dynein motor, as microtubule-disrupting drugs strongly reduced the transport of HSV-1 capsids to the nucleus [21]. The HSV-1 capsid protein VP26 [26], the inner nuclear membrane protein pUL34 [27], the tegument protein US11 [28], and the helicase pUL9 [29] have been found to interact with cytoplasmic kinesin or dynein and are crucial for mediating viral intracellular transport. More recent research has indicated that HSV-1 ICP5, the major capsid protein can interact with the dynein light chain, although this putative interaction needs to be confirmed in infected cells [30]. Hsp90 is involved in nuclear localization, vRNP complex formation, and viral RNA synthesis within nucleus in other virus infection such as influenza virus infection [31]–[33]. Hsp90 may actively participate in multiple stages of HSV-1 infection, including intracellular transport, nuclear translocation, and viral DNA replication within infected nucleus. It has been observed that Hsp90 chaperone system, including Hsp70 and Hsp40, is necessary for HSV-1 infection and helps to localize HSV-1 DNA polymerase to the nucleus [34], the mechanism by which Hsp90 involves in HSV-1 nuclear transport remains unclear. In particular, Hsp90 and other molecular chaperones, such as Hsc70, Hsp70, and Hsp40, are recruited to nuclear domains and may contribute to the promotion of viral protein folding and transport [35]. Hsp70 specifically binds to tubulin and other MT-associated proteins (MAPs) to enhance MT polymerization [36]. Another chaperone, Hsp27, is also rapidly reorganized and modified in response to HSV-1 infection. The subcellular localization of Hsp27 is similar to that of Hsp90 [37], and Hsp27 also associates with the MT system [38]. These findings suggest that molecular chaperones play important roles in intracellular transport of HSV-1 capsids. In the present study, we revealed that Hsp90 is required for nuclear transport of HSV-1 capsid protein ICP5. Hsp90 is rapidly induced in response to HSV-1 infection and is then translocated into and enriched in the nucleus of infected cells coincides with the major HSV-1 capsid protein ICP5. Simultaneously, HSV-1 infection facilitates acetylation of α-tubulin and upregulated Hsp90 is recruited to interact with acetylated α-tubulin. Treatment of Hsp90 inhibitors reduces virus-induced acetylation of α-tubulin, the interaction of Hsp90-tubulin, and viral ICP5 nuclear transport. It has been reported that Hsp90 binds to tubulin and inhibits MT formation [39]. The interaction of Hsp90 with MTs depends on the level of tubulin acetylation, which also stimulates the binding and signaling functions of Hsp90 client proteins [25].

It has been reported that the HSV-1 tegument protein VP22 can induce hyperacetylation and stabilization of MTs during both transfection and infection [40], suggesting that VP22 is one of the viral inducers for stimulating the acetylation of α-tubulin. Tubulin acetylation is known to be regulated by HDAC6, a class II histone deacetylase [41]. HDAC6 interacts with Hsp90 on MTs to form HDAC6/heat shock factor 1 (HSF1)/Hsp90 complex under unstressed condition [42], and Hsp90 chaperone activity is regulated by reversible acetylation and controlled by HDAC6 [43]. Our results demonstrated that Hsp90 inhibition significantly reduces HSV-1-induced acetylation of tubulin, suggesting that Hsp90 activity is involved in the regulation of acetylation of tubulin during HSV-1 infection. We can speculate that acetylated tubulin induced by HSV-1 infection not only stabilizes MTs but is also important for strengthening the chaperone function of Hsp90 in the recruitment of viral capsids to MTs, thus facilitating viral capsid nuclear translocation. Moreover, we infer that HDAC6 plays an important role in regulating the interaction between Hsp90 and acetylated α-tubulin. The roles of HDAC6 and Hsp90 in HSV-1 intracellular capsid translocation deserve further investigation to elucidate.

Emergence of drug resistant strain is the main hurdles in the development of effective anti-HSV agents. Antiviral compounds that target viral proteins easily generate viral escape mutations, resulting in drug resistance [10]. Hsp90 is a host protein that is required for HSV-1 replication and the inhibitors of Hsp90 is generally considered to present a low risk of generating drug-resistant viruses. Additionally, the role of Hsp90 in inflammation and cellular innate immune defense pathways make this protein a promising target for an antiviral approach [44]. Hsp90 regulates the activation of interferon regulatory factor 3 and TBK-1 stabilization to facilitate Sendai virus infection [45] and plays a role in antigen cross-presentation during lymphocytic choriomeningitis virus infection [46]. In agreement with the findings of previous reports regarding the anti-HSV activity of GA [12], in the current study, the Hsp90 inhibitors BJ-B11 and 17-AAG presented significant antiviral activity against HSV-1, with IC_50_ values less than that of ACV. Previously GA has been demonstrated to inhibit viral replication, release, and restore the cell cycle. However, specific role of Hsp90 in HSV-1 early infection and whether GA has an antiviral effect during HSV-1 early infection have not been illuminated. Herein, our results show that Hsp90 plays a critical role in viral capsid protein translocation and novel Hsp90 inhibitor BJ-B11 exhibits potent antiviral effect by reducing viral nuclear transport.

In conclusion, we are the first to reveal that Hsp90, by interacting with acetylated α-tubulin, plays a crucial role in promoting viral nuclear transport. Hsp90 inhibitors suppressed drug-resistant HSV-1 replication by interfering with the interaction between Hsp90 and α-tubulin, thereby inhibiting capsid nuclear translocation and replication. The present study provides novel insight into the mechanism by which Hsp90 mediates viral capsids nuclear translocation and into the anti-HSV mechanism of Hsp90 inhibitors.
